# Acute oral toxicity evaluation of extracts of *Hydrocotyle sibthorpioides* in wister albino rats as per OECD 425 TG

**DOI:** 10.1016/j.toxrep.2019.04.001

**Published:** 2019-04-04

**Authors:** Iswar Hazarika, K.M. Geetha, P. Sivakami Sundari, Divya Madhu

**Affiliations:** a Department of Pharmacology, College of Pharmaceutical Sciences, Dayananda Sagar University, Shavige Malleshwara Hills, Kumaraswamy Layout, Bengaluru, Karnataka, India -560078; b Department of Pharmacognosy, College of Pharmaceutical Sciences, Dayananda Sagar University, Shavige Malleshwara Hills, Kumaraswamy Layout, Bengaluru, Karnataka, India -560078

**Keywords:** Hydrocotyle sibthorpioides, LD_50_, Acute oral toxicity studies, Apiaceae

## Abstract

*Hydrocotyle sibthorpioides* (Family: Apiaceae) is a medicinal plant and is been a part of traditional cuisine. Hence, an acute toxicity study was performed to confirm its safety profile. Female rats (n=5) were randomly divided into 5 groups. One group served as the control and the other groups were been administered with the petroleum ether, chloroform, methanolic and aqueous extract of Hydrocotyle sibthorpioides at 2000 mg/kg body weight. Blood was collected at certain intervals to measure the haematological and biochemical parameters. The vital organs were isolated and preserved for histopathological examinations.

The result suggested that the LD_50_ of all the extracts are greater than 2000 mg/kg body weight. There was no significant change in body weight, Behaviour, Renal Function test, Liver function test and Lipid profile. In haematological evaluation, there was an increase in RBC count and haemoglobin production post administration of methanolic and aqueous extract. The study suggested that the LD_50_ of the plant is greater than 2000 mg/kg body weight of rats in all the extracts.

## Highlights

• Toxicity was studied for *Hydrocotyle sibthorpioides*.
• LD_50_ was found to be greater than 2000 mg/kg of body weight.
• Petroleum ether extract showed increase in somatomotor activity for short time.
• Methanolic and aqueous extract exhibited some change in Haematological parameters.

## Graphical abstract

**Figure:**
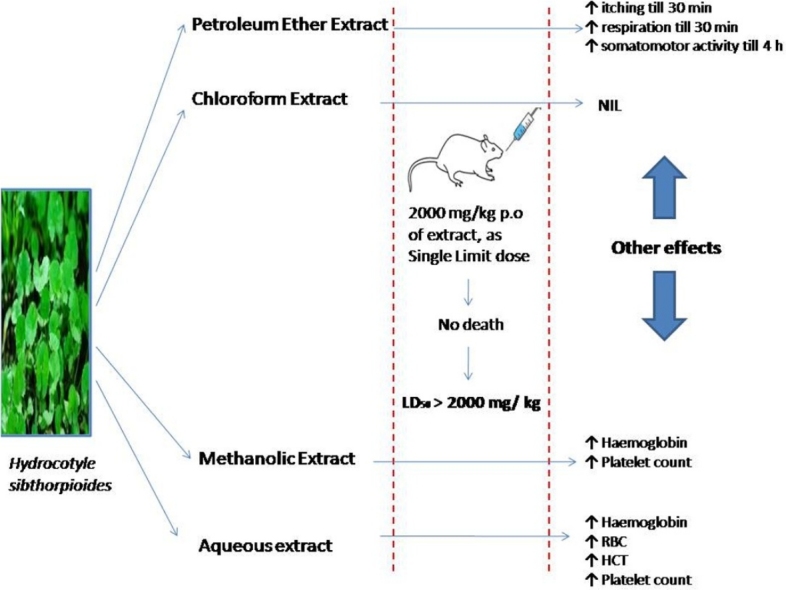

## 1 Introduction

Traditional medicines play a major role in the rural areas in-spite of the development in synthetic and semi-synthetic drugs for the treatment of different ailments. The utility of these herbal medicines is showing a tremendous shift towards the overuse [1]. As father of toxicology, Paracelsus said “All substances are poisons; there is none which is not a poison. It is the right dose which differentiates remedy from poison” [2], it is the need of this hour is to conduct research on the safety profile of the medicinal plants. The medicines of plant origin is expected to have very less toxicity but certain medicinal plant used in traditional medicines are been reported to exhibit toxic effect [3], [4].

*Hydrocotyle sibthorpioides* Lam. belonging to the family Apiaceae is one such widespread and perennial herb which has been used traditionally by the people of India and China in different ailments. In India it has been used as a brain tonic, fever, edema, soothing pain and detoxication [5], [6], [7]. The plant is also used in Chinese medicine for the treatment of immune disorders and Hepatitis [8]. It is reported that it is efficient in the treatment of rheumatalgia, dysentery, jaundice, and exerted a potent inhibitory effect on the growth of tumours [9]. Recently, the *in vitro* and *in vivo* antiviral properties of *H. sibthorpioides* against hepatitis B virus replication have been demonstrated [10]. Moreover, this plant is been used in different traditional cuisines of Assam. So, there is a far above the ground prevalence of this plant to induce toxicity. Therefore, the study was designed to explore the acute oral toxicity profile of *Hydrocotyle sibthorpioides.*

## 2 Materials and methods

### 2.1 Collection of plants

The whole plant of *Hydrocotyle sibthorpioides* was collected in the month of February- April 2018 from the fields of Kalabari, District-Biswanath chariali, PIN-784178, Assam, India and was identified as *Hydrocotyl sibthorpioides* Lam. (family Apiaceae) by a taxonomist at Botanical Survey of India, Eastern regional centre, Shillong with Authentication letter No. *BSI/ERC/Tech/2018/106 dated 17/05/2018*. The plants were shade dried and were ground to fine powder.

### 2.2 Preparation of crude extracts

The fine powder of *Hydrocotyl sibthorpioides* (1700 g) was subjected to successive solvent extraction by soxhlet apparatus using Petroleum ether, followed by Chloroform and Methanol as solvents. Aqueous extract was prepared post methanolic extraction by cold macerating for 7 days and 10 ml of ethanol. The extracts obtained were concentrated in a rotary evaporator in reduced pressure.

### 2.3 Preparation of dose

The different extracts of *Hydrocotyl sibthorpioides* viz. Petroleum Ether Extract(PEHS), Chloroform Extract(CEHS), Methanolic Extract(MEHS) and Aqueous Extract (AEHS) were suspended with 0.3% Carboxymethyl cellulose (CMC).

### 2.4 Preliminary phytochemical studies

The extracts were screened for the presence of various phytoconstituents according to the procedure described by Khandelwal [11].

### 2.5 Animals and approval from animal ethical committee

Healthy nulliparous and non-pregnant female wister Albino rats (150–200 g m) between 8–12 weeks were used for all the experiments in the present study. The animals were maintained under standard husbandry conditions in the animal house of ‘College of Pharmaceutical Sciences, Dayananda Sagar University’ (temperature 25 ± 2 °C) in a natural light-dark cycle and fed with standard rodent diet and water ad libitum. Ethical committee clearance was obtained from IAEC (Institutional Animal Ethics Committee) of CPCSEA (Ref. No. DSU/PhD/IAEC/11/2017–2018).

### 2.6 Acute toxicity assay

**Fig. 1:**
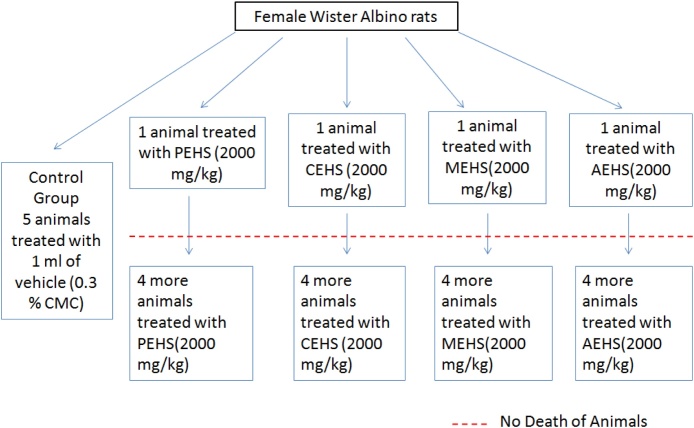
Grouping of animals for acute toxicity studies.

To allow for acclimatisation to the laboratory conditions, the rats were selected randomly, marked to allow individual recognition, and kept in their cages for at least 5 days prior to dosing. The animals were kept without food for overnight prior dosing but had access to water. Acute toxicity study was performed with a Limit test at 2000 mg/kg p.o. as single dose for all the extracts as shown in the Fig. 1. The dose was administered to the animal based on their body weight. The animals were closely observed for first 30 min, then for 4 h. Food was provided after 1–2 h of dosing. After survival of the treated animal 4 more animals were treated with the same dose.

A control group of rats (n = 5) were administered with 0.3% CMC in the same volume of that of the treated group. All the groups were closely observed for 6 h and then at a regular interval for 14 weeks. Survived animals were observed for other toxic effects. The weight of the animals was monitored from the beginning of the experiment and the blood samples were collected by cardiac puncture under anaesthesia with chloroform and serum was separated for biochemical and haematological evaluations. After the end of the experiment the animals were sacrificed by cervical dislocation and vital organs were excised, weighed and preserved in 10% formalin for histopathological evaluation.

### 2.7 Haematological analysis

The blood samples from animals (both treated and vehicle control groups) were collected in EDTA containing tubes for haematological study. CBC parameters, hemoglobin (Hb), total RBC, packed cell volume (PVC), mean corpuscular volume (MCV), mean corpuscular haemoglobin (MCH), mean corpuscular hemoglobin concentration (MCHC), platelet count, white blood cells (WBC) count, neutrophils (N), lymphocytes (L), monocytes (M), and eosinophils (E) were determined.

### 2.8 Biochemical analysis

Different biochemical parameters were measured using Randox kits in a biochemical analyser. The parameters observed for renal function are Creatinine, Urea. For liver function parameters observed are aspartate aminotransferase (AST), alanine aminotransferase (ALT), alkaline phosphate, bilirubin, albumin and globulins.

Lipid Profile like Cholesterol, Triglyceride, high density lipoprotein (HDL), very low density lipoprotein (VLDL), low density lipoprotein (LDL) and Cholesterol/ HDL ratio were observed.

### 2.9 Histopathological study

The vital organs isolated from sacrificed rats were fixed in 10% formalin, then after processing embedded in paraffin wax. Paraffin sections were made at 5 mm and stained with hematoxylin and eosin. The slides were studied under a light microscope and captured the magnified images of tissues structure for further study.

### 2.10 Statistical analysis

Experimental results were presented as mean ± SEM and the statistical significance between the groups was analyzed by means of one way ANOVA followed by Dunnet’s multiple comparison test P ≤ 0.05 was considered as statistically significant.

## 3 Results

### 3.1 Phytochemical investigation

The preliminary phytochemical investigation of the extracts of *Hydrocotyl sibthorpioides* revealed the presence of different chemicals which are been listed in Table 1.

**Table 1:** Phytochemical investigation of *Hydrocotyl sibthorpioides* extracts.

PEHS	CEHS	MEHS	AEHS
Phytosterols & Triterpenoides	Phytosterols & Triterpenoides	Tannins & Phenolic Compounds	Carbohydrates
Glycosides: – Cardiac – Coumarin	Glycosides: – Cardiac – Coumarin	Glycosides: – Cardiac – Flavonoides	Glycosides: – Cardiac – Anthraquinone
Alkaloids	-Fixed oils and fats	Flavonoides	Saponins
Volatile oil		Saponins	
Fixed oils and fats			

PEHS: Petroleum ether extract of *Hydrocotyl sibthorpioides*; CEHS: Chloroform extract of *Hydrocotyl sibthorpioides*; MEHS: Methanolic extract of *Hydrocotyl sibthorpioides*; AEHS: Aqueous extract of *Hydrocotyl sibthorpioides*;

### 3.2 Acute oral toxicity assay

Limit test with dose of 2000 mg/kg body weight of rats exhibited no mortality with different extracts of *Hydrocotyl sibthorpioides* by using 0.3% of CMC as vehicle. The test animals were observed for 1st 30 min. and then for 4 h. Observations were recorded on a regular basis for 14 days. The results are as follows:

#### 3.2.1 Behavioural pattern and body weight

**Fig. 2:**
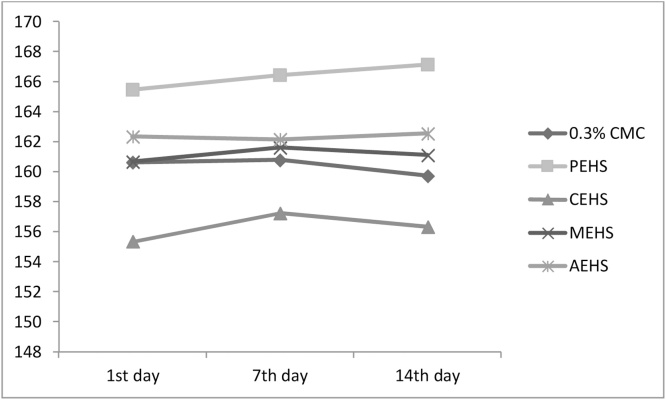
Effect of *Hydrocotyl sibthorpioides* different extracts on body weight of rats in acute toxicity studies. PEHS: Petroleum ether extract of *Hydrocotyl sibthorpioides*; CEHS: Chloroform extract of *Hydrocotyl sibthorpioides*; MEHS: Methanolic extract of *Hydrocotyl sibthorpioides*; AEHS: Aqueous extract of *Hydrocotyl sibthorpioides*; Values are presented as mean ± SEM; N = 5.

The body weights of all the animals, both in control and the treated animals are shown in Fig. 2. There was no significance change in the body weight after the treatment of different extracts.

Behavioural observations for the group treated with PEHS showed itching, increased respiration and increase in somatomotor activity in the first 30 min.. The somatomotor activity was present till the 4 h of the study which was abolished post 24 h of treatment. All other parameters like Eyes, Faeces consistency, Fur and skin, Salivation, Urine colour and sleep were normal during the study. The detailed observations are showed in Table 2.

**Table 2:** Effect of *Hydrocotyl sibthorpioides* different extracts on behaviour of rats in acute toxicity studies.

Parameters	Observations of vehicle control and *Hydrocotyl sibthorpioides*extract treated groups																													
	30 Min					4 h					24 h					48 h					7 days					14 days				
	0.3% CMC	PEHS	CEHS	MEHS	AEHS	0.3% CMC	PEHS	CEHS	MEHS	AEHS	0.3% CMC	PEHS	CEHS	MEHS	AEHS	0.3% CMC	PEHS	CEHS	MEHS	AEHS	0.3% CMC	PEHS	CEHS	MEHS	AEHS	0.3% CMC	PEHS	CEHS	MEHS	AEHS
Coma	A	A	A	A	A	A	A	A	A	A	A	A	A	A	A	A	A	A	A	A	A	A	A	A	A	A	A	A	A	A
Convulsions & tremors	A	A	A	A	A	A	A	A	A	A	A	A	A	A	A	A	A	A	A	A	A	A	A	A	A	A	A	A	A	A
Eyes	N	N	N	N	N	N	N	N	N	N	N	N	N	N	N	N	N	N	N	N	N	N	N	N	N	N	N	N	N	N
Faeces consistency	N	N	N	N	N	N	N	N	N	N	N	N	N	N	N	N	N	N	N	N	N	N	N	N	N	N	N	N	N	N
Fur & Skin	N	N	N	N	N	N	N	N	N	N	N	N	N	N	N	N	N	N	N	N	N	N	N	N	N	N	N	N	N	N
Itching	A	P	A	A	A	A	A	A	A	A	A	A	A	A	A	A	A	A	A	A	A	A	A	A	A	A	A	A	A	A
Mortality	A	A	A	A	A	A	A	A	A	A	A	A	A	A	A	A	A	A	A	A	A	A	A	A	A	A	A	A	A	A
Mucous membrane	N	N	N	N	N	N	N	N	N	N	N	N	N	N	N	N	N	N	N	N	N	N	N	N	N	N	N	N	N	N
Respiration	N	↑	N	N	N	N	N	N	N	N	N	N	N	N	N	N	N	N	N	N	N	N	N	N	N	N	N	N	N	N
Salivation	N	N	N	N	N	N	N	N	N	N	N	N	N	N	N	N	N	N	N	N	N	N	N	N	N	N	N	N	N	N
Sleep	N	N	N	N	N	N	N	N	N	N	N	N	N	N	N	N	N	N	N	N	N	N	N	N	N	N	N	N	N	N
Somatomotor activity & behavior pattern	N	↑	N	N	N	N	↑	N	N	N	N	N	N	N	N	N	N	N	N	N	N	N	N	N	N	N	N	N	N	N
Urination (colour)	N	N	N	N	N	N	N	N	N	N	N	N	N	N	N	N	N	N	N	N	N	N	N	N	N	N	N	N	N	N

PEHS: Petroleum ether extract of *Hydrocotyl sibthorpioides*; CEHS: Chloroform extract of *Hydrocotyl sibthorpioides*; MEHS: Methanolic extract of *Hydrocotyl sibthorpioides*; AEHS: Aqueous extract of *Hydrocotyl sibthorpioides*; A- Absent; N- Normal; ↑- Increase.

#### 3.2.2 Hematological analysis

**Fig. 3:**
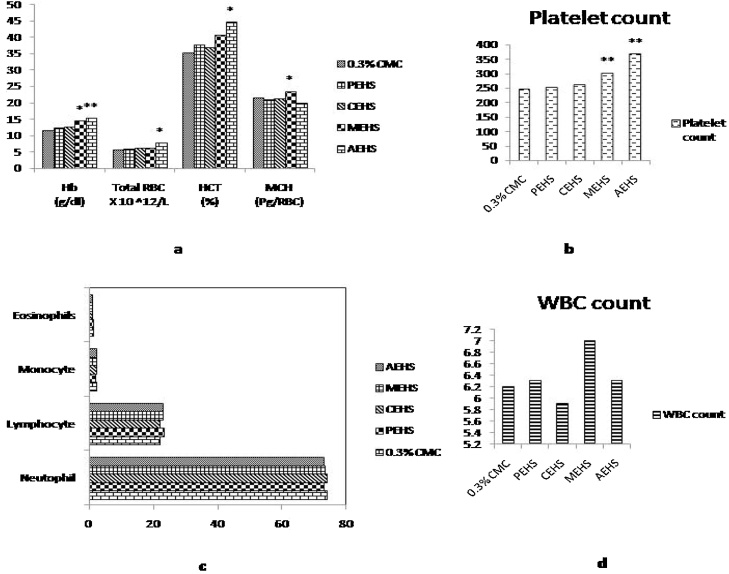
Effect of extracts given at Limit dose (2000 mg/kg bw of rats) in Hematological profile. Fig. 3a shows the effects of different extracts on Haemoglobin count (Hb), Total RBC, Hematocrit (HCT) and Mean Corpuscular volume (MCH); Fig. 3b shows the effect of extracts on blood platelets; Fig. 3c and d shows the effects of extracts on differential leukocyte count and total WBC count respectively. PEHS: Petroleum ether extract of *Hydrocotyl sibthorpioides*; CEHS: Chloroform extract of *Hydrocotyl sibthorpioides*; MEHS: Methanolic extract of *Hydrocotyl sibthorpioides*; AEHS: Aqueous extract of *Hydrocotyl sibthorpioides*; Values are presented as mean ± SEM; N = 5. *p < 0.05 when compared with the vehicle group (0.3% Carboxymethyl cellulose gel).

The results of the hematological analysis are given in Fig. 3. It can be seen that there was no remarkable alterations in any parameters in PEHS and CEHS treated group when compared with the control. Nevertheless, there was a significant raise in Hb count in MEHS (P < 0.05) and AEHS (P < 0.01) treated groups as compared to the control group. Total RBC count (P < 0.05), HCT (P < 0.05) and Platelet count (P < 0.01) was seen to be significant in Aqueous extract treated group as compared to the control. Platelet count (P < 0.01) and MCH (P < 0.05) was seen to be significantly high in MEHS treated group as compared to the control.

#### 3.2.3 Biochemical analysis

**Fig. 4:**
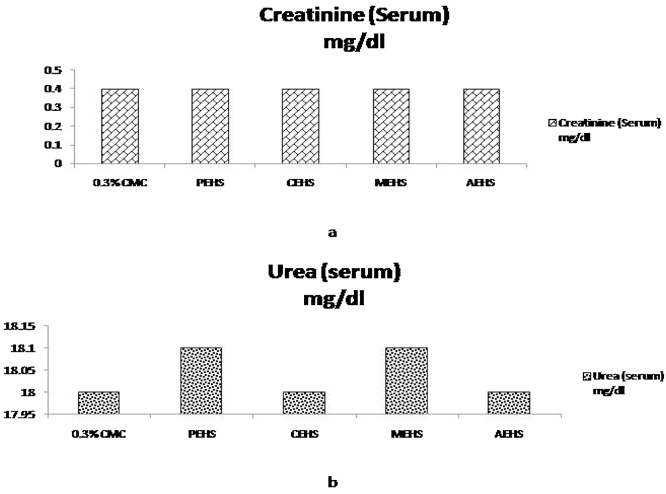
Effect of extracts given at Limit dose (2000 mg/kg bw of rats) in Renal function test. Fig 4a & Fig 4b shows the serum Creatinine level & Serum Urea level respectively; PEHS: Petroleum ether extract of *Hydrocotyl sibthorpioides*; CEHS: Chloroform extract of *Hydrocotyl sibthorpioides*; MEHS: Methanolic extract of *Hydrocotyl sibthorpioides*; AEHS: Aqueous extract of *Hydrocotyl sibthorpioides*; Values are presented as mean ± SEM; N = 5. *p < 0.05 when compared with the vehicle group (0.3% Carboxymethyl cellulose gel).

**Fig. 5:**
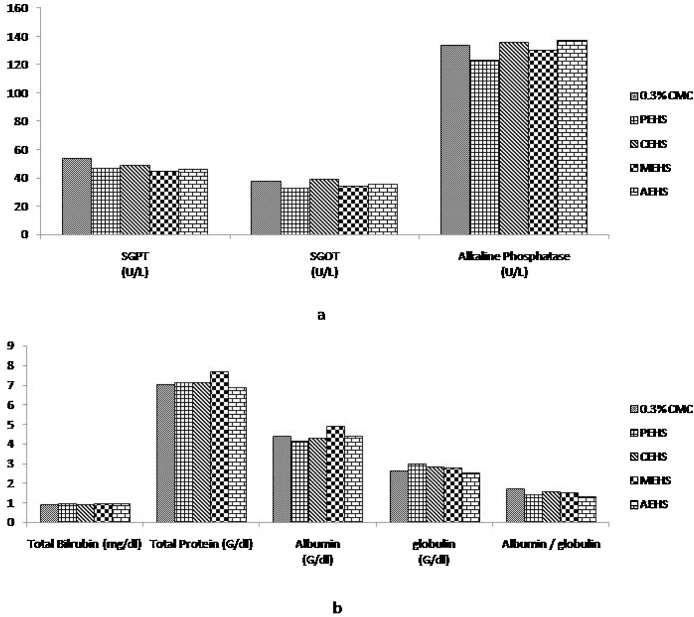
Effect of extracts given at Limit dose (2000 mg/kg bw of rats) in Liver function test; Fig. 5a shows the effect of the extracts on SGPT, SGOT and Alkaline Phosphatase; Fig. 5b shows the effect of the extracts on Total Bilrubin, Total Protein, Albumin, Globulin and Albumin to Globulin ratio. PEHS: Petroleum ether extract of *Hydrocotyl sibthorpioides*; CEHS: Chloroform extract of *Hydrocotyl sibthorpioides*; MEHS: Methanolic extract of *Hydrocotyl sibthorpioides*; AEHS: Aqueous extract of *Hydrocotyl sibthorpioides*; Values are presented as mean ± SEM; N = 5. *p < 0.05 when compared with the vehicle group (0.3% Carboxymethyl cellulose gel).

**Fig. 6:**
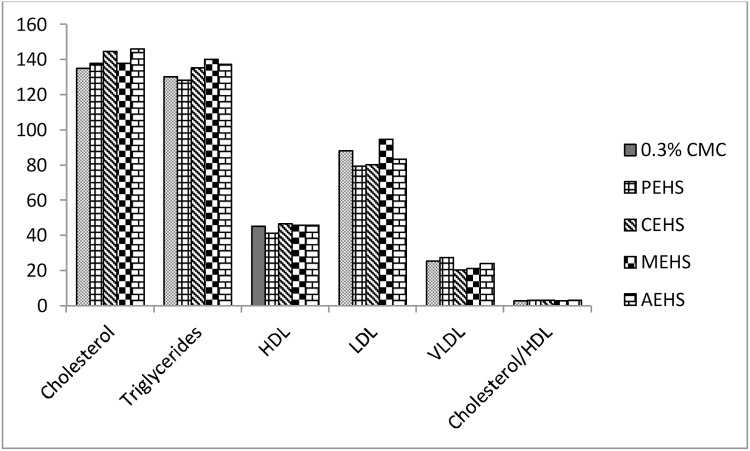
Effect of extracts given at Limit dose (2000 mg/kg bw of rats) in Lipid profile. PEHS: Petroleum ether extract of *Hydrocotyl sibthorpioides*; CEHS: Chloroform extract of *Hydrocotyl sibthorpioides*; MEHS: Methanolic extract of *Hydrocotyl sibthorpioides*; AEHS: Aqueous extract of *Hydrocotyl sibthorpioides*; Values are presented as mean ± SEM; N = 5 *p < 0.05 when compared with the vehicle group (0.3% Carboxymethyl cellulose gel).

The various biochemical parameters post limit dose treatment showed no significant change in the Renal function test (Fig. 4), Liver function Test (Fig. 5) and Lipid profile (Fig. 6).

#### 3.2.4 Histopathological studies

**Fig. 7:**
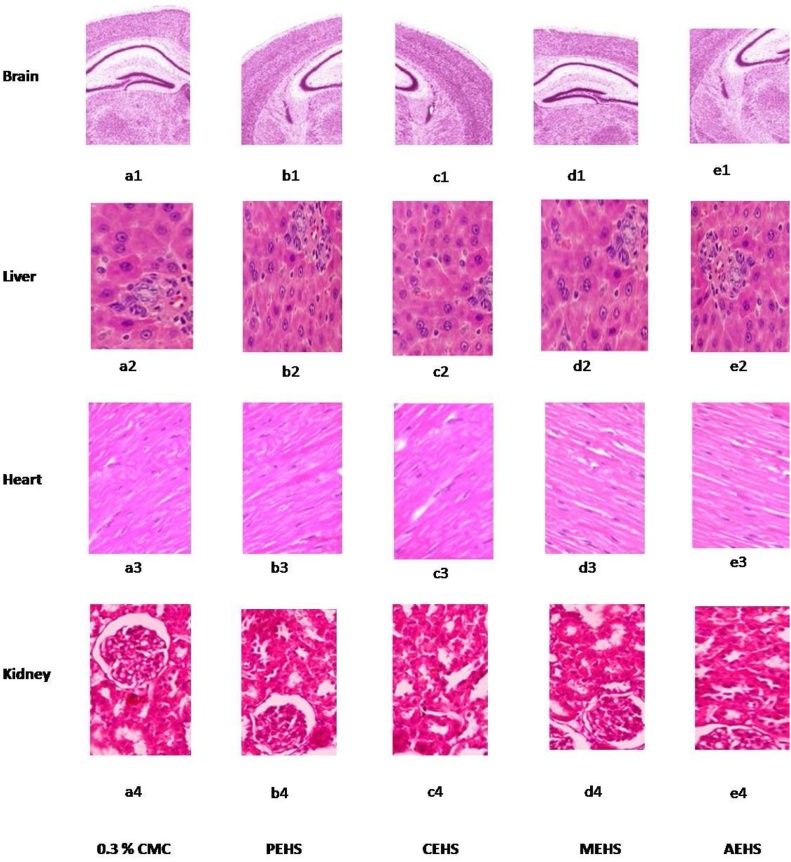
Effect of extracts given at Limit dose (2000 mg/kg bw of rats) on the Histology of the vital organs. PEHS: Petroleum ether extract of *Hydrocotyl sibthorpioides*; CEHS: Chloroform extract of *Hydrocotyl sibthorpioides*; MEHS: Methanolic extract of *Hydrocotyl sibthorpioides*; AEHS: Aqueous extract of *Hydrocotyl sibthorpioides.*

The histopathological report of all the vital organs viz. Brain, heart, liver and kidney suggested no change in any treated group as compared to the control. (Fig. 7)

## 4 Discussion

Medicinal plants are been used since centuries to treat different ailments. One such medicinal plant is *Hydrocotyl sibthorpioides,* which has been in used since centuries by the people of India and China. This plant is also an integral part of the traditional cuisine of Assamese people. These may lead to toxicity of the plant constituents, as it is the dose that makes the drug a poison. Hence, this project was designed to check the toxic effect of the plant by oral route using 425 toxicity guidelines.

The toxic outcomes can be measured by the clinical signs and symptoms among other toxicity indicators [12]. No animals were found death after the administration of limit dose of 2000 mg/kg body weight of rat while behavioural studies like respiration, increased somatomotor activity and itching were been observed during the first 30 min after the petroleum ether extract administration. No significant change in the body weight was been observed during the course of the study but the food and water intake was normal. It suggested that the normal processing of all the nutrients like carbohydrate, proteins and fats are been metabolised appropriately within the body as these are the nutrients that play a major role in physiological function [13], [14], [15].

Brain, Liver, Kidney and heart are the major vital organs of the body that are been affected by the toxic substance [16].When animals were sacrificed at the end of study, there were no lesions found on histological examination of Brain, heart, kidney and liver in comparison with vehicle control group. (Fig. 7) Chemicals are divided into five groups on the basis of LD_50_ according to the globally harmonized classification system [17]. Therefore, all the extracts of *Hydrocotyl sibthorpioides* can be put into group 5 (LD_50_ > 2000 mg/kg), which is the class of lower toxicity.

The health status of the body can be evaluated by other biological parameters including serum biomarkers measurement. Liver injury can be assessed by the elevated SGPT, SGOT and total protein level [18], [19], [20], [21]. There was no significant change in the SGPT, SGOT, albumin, globulin or total proteins. (Fig. 5) The report supports the finding of the histological reports.

Multiple hyperlipidemias are always secondary to some factors which include drugs [22]. The results of our study (Fig. 6) suggested that there is no significant change in the lipid profile post administration of limit dose of 2000 mg/kg of *Hydrocotyl sibthorpioides* extracts suggesting it’s no influence on lipid profile.

Renal impairment is indicated by the elevation of serum creatinine and urea [23]. In the present study there is no significant change in serum creatinine and urea, which can be an indicative observation for a very safe plant for its use in traditional cuisine.

Hematological parameters are perceptive markers of the physiological changes in retort to any toxic stress or environmental pollutant in animals [24]. Blood platelet plays a vital role in Haemostasis [25]. This study showed a remarkable increase of platelets by methanolic and aqueous extract (Fig. 3). The improved platelet level could be an added advantage for the treatment of Dengue by *Hydrocotyl sibthorpioides* [26]. The Haematological profile on the other hand suggested that there was an increase in the RBC count and Haemoglobin count post treatment of AEHS and MEHS which suggest that there is some influence of the extract on the haematopoiesis pathway. Statistically there is no significant change in WBC count and differential WBC count which suggest that there is no cellular inflammation process.

## 5 Conclusion

In the light of finding of acute toxicity studies as per 425 it can be concluded that all the plant extracts used in the study are comparatively very safe with LD_50_ > 2000 mg/Kg (Group 5 of toxicity class as per GHS). However, preliminary results suggested that it should be evaluated for chronic toxicity studies post repeated administrations to ensure the safety of *Hydrocotyl sibthorpioides*.
